# Leisure-Time Physical Activity Participation in Middle-Aged and Older Adults With a Spinal Cord Injury in Australia

**DOI:** 10.3389/ijph.2024.1607276

**Published:** 2024-07-03

**Authors:** Laura Stendell, Peter W. Stubbs, Kris Rogers, Arianne P. Verhagen, James W. Middleton, Glen M. Davis, Mohit Arora, Ruth Marshall, Timothy Geraghty, Andrew Nunn, Camila Quel de Oliveira

**Affiliations:** ^1^ Discipline of Physiotherapy, Graduate School of Health, Faculty of Health, University of Technology Sydney, Sydney, NSW, Australia; ^2^ Graduate School of Health, Faculty of Health, University of Technology Sydney, Sydney, NSW, Australia; ^3^ John Walsh Centre for Rehabilitation Research, Northern Sydney Local Health District, St Leonards, NSW, Australia; ^4^ The Kolling Institute, Faculty of Medicine and Health, The University of Sydney, Sydney, NSW, Australia; ^5^ Discipline of Exercise and Sport Science, School of Health Sciences, Faculty of Medicine and Health, The University of Sydney, Sydney, NSW, Australia; ^6^ Faculty of Health and Medical Sciences, University of Adelaide, Adelaide, SA, Australia; ^7^ South Australian Spinal Cord Injury Service, Central Adelaide Local Health Network, Adelaide, SA, Australia; ^8^ The Hopkins Centre, Metro South Health and Menzies Health Institute Queensland, Griffith University, Brisbane, QLD, Australia; ^9^ Queensland Spinal Cord Injuries Service, Division of Rehabilitation, Princess Alexandra Hospital, Brisbane, QLD, Australia; ^10^ Victorian Spinal Cord Service, Austin Health, Heidelberg, VIC, Australia

**Keywords:** leisure-time physical activity, spinal cord injury, middle-aged, older adult, physical activity

## Abstract

**Objectives:**

Adults with spinal cord injury (SCI) are often sedentary, increasing their risk of cardiometabolic diseases. Leisure-time Physical Activity (LTPA) is physical activity completed during recreation time for enjoyment. We aimed to quantify LTPA in people ≥45 years with SCI and to explore its relationship with participants’ characteristics.

**Methods:**

This is a secondary analysis on a subset of the Australian International SCI Survey in participants ≥45 years, at least 12 months post-injury. We described levels of LTPA and used multivariable regressions to estimate the associations between participant characteristics and LTPA.

**Results:**

Of 1,281 participants (mean age: 62.7 years, mean time since injury: 18.7 years; 74% males) 44% reported no participation in LTPA. The average LTPA participation was 197 (SD 352) minutes per week (median: 50). Females (β = −62.3, 95% CI [−112.9, −11.7]), and participants with non-traumatic injuries (β = −105.2, 95% CI [−165.9, −44.6]) performed less LTPA. Time since injury was not associated with moderate-to-heavy LTPA (LR: Probability > F = 0.785).

**Conclusion:**

LTPA promotion in the SCI population ≥45 years focusing on females and non-traumatic injuries is warranted.

## Introduction

People with spinal cord injury (SCI) have very low levels of physical activity participation and are among the most inactive populations with physical disabilities [1]. The reasons for low engagement in physical activity vary but include loss of muscle function and strength and reduced energy expenditure [2], reduced mobility and fitness, pain, and environmental barriers, such as lack of accessible gyms, specialised programs or knowledgeable health professional in the community [3–5]. Low levels of physical activity contribute to an increased risk of cardiometabolic diseases, weight gain, pressure injuries, and infections, which may lead to frequent hospitalisations [6]. With an overall aging population and advances in medical care, the life expectancy of people living with SCI has increased, and there has been a shift in the demographic profile towards an older population sustaining and living with a SCI [7–9]. It is estimated that 20,800 people are living in Australia with an SCI, with approximately 175 new injuries each year [10]. The age-specific rate is highest for those aged 55–64 at 11.2 cases per million, closely followed by 10.1 cases per million for those aged 65–74 [10]. The main causes of traumatic injury are motor vehicle accidents (46%) and closely followed by falls (36%) [10]. Older people with SCI are usually less active than younger people and have an increased risk of developing secondary health conditions related to reduced physical activity [11, 12].

The World Health Organization (WHO) physical activity guidelines for adults with physical disabilities recommends that for cardiovascular disease risk-reduction, people need to participate in 150–300 min of moderate-intensity aerobic physical activity per week, or 75–150 min of vigorous aerobic physical activity per week, or a combination of the two, as well as strength-training at moderate intensity for all major muscle groups on 2 or more days per week [13]. Additionally, the physical activity guidelines for adults with SCI, recommend participation in at least 20 min of moderate-to-heavy intensity aerobic exercise twice a week plus three sets of strength exercises for each major functioning muscle group, at a moderate-to-heavy intensity, twice a week (for cardiorespiratory and muscle strength benefits). In addition, three times per week, including at least 30 min moderate-to-heavy intensity aerobic exercise, advised for cardiometabolic health benefits [14]. Despite these recommendations, research shows that between 27% [15] and 50% [16] of the SCI population are inactive, and those who engage in physical activity often do not meet the recommended levels of either guidelines particularly older adults [3, 11].

The risk of developing secondary cardiovascular disease and comorbidities might be reduced by participation in regular exercise and leisure-time physical activity (LTPA) [17]. LTPA is a physical activity undertaken in a person’s leisure-time or free time, such as playing sports, wheeling, walking, gardening, exercising at a gym and other recreational activities [18]. Further, increased LTPA positively impacts wellbeing, psychological health, and health-related quality of life in individuals with SCI [19–21]. Despite prior research on LTPA in the SCI population, limited evidence exists for middle-aged and older individuals with SCI regarding their participation, intensity, and duration of LTPA [11, 22]. A Swedish cohort study showed that many individuals with SCI over 50 years, and at least 10 years post-injury, did not meet minimum physical activity requirements necessary to achieve health benefits, with age and wheelchair use predicting low engagement [11].

In this study, we focussed on individuals with SCI who were ≥45 years, to represent effects of premature aging on multiple body systems [23]. At this age, many people in the general population begin to experience physical, psychological, and social changes associated with the aging process [24]. Defining “middle-aged and older” as ≥45 years is supported by multiple large-scale cohort studies that investigated the effects of aging in the general population [25–28]. These studies have provided important insights into the early stages of aging and helped to identify risk factors for age-related disabilities. This age definition allowed the investigation of physical activity participation in those experiencing the early effects of aging and acknowledges that, due to autonomic dysfunction, cardiometabolic and musculoskeletal changes associated with SCI [23, 29–31], people with SCI experience the effects of aging earlier than the general population [23]. Further, this age definition allows for future comparisons with sizeable studies in the general population. Therefore, the primary aim of this study was to describe the amount of LTPA performed by individuals with SCI ≥45 years living in Australia. The main objective was to describe levels of LTPA participation using the modified version of the Physical Activity Scale for Individuals with Physical Disabilities (PASIPD). The secondary aim was to investigate the relationships between LTPA and injury and demographic characteristics, with the objectives: 1) to determine the potential associations between LTPA and age, gender, completeness of injury and level of injury and 2) to explore the associations between time since injury and levels of LTPA.

## Methods

Secondary analyses of the Australian arm of the International Spinal Cord Injury (Aus-InSCI) Community Survey was performed [32, 33]. This is a large cross-sectional survey that included questions about LTPA participation in Australians with SCI of all ages. The Aus-InSCI survey was conducted in 2018 to capture the lived experience of people living with SCI in the community in many areas including health, wellbeing, activity, independence, participation, leisure-time physical activity and sociodemographic characteristics [32, 33]. The Aus-InSCI survey was part of a larger international project investigating the lived experience of people with SCI across 22 countries. The design of the Aus-InSCI study and its cohort description have been published elsewhere [32]. The authors acknowledge that as this is a retrospective analysis, the data does not represent the lived experience of people with SCI during the COVID-19 pandemic.

The Aus-InSCI study was approved by the Northern Sydney Local Health District HREC (HREC/16/HAWKE/495) and Australian Institute of Health and Welfare Ethics Committee (EO2017/1/341). The ethical approval for this study was obtained from the Human Ethics Committee at the University of Technology Sydney (ETH22-7573).

### Participants

Inclusion criteria for the Aus-InSCI study were adults aged ≥18 years old, community-dwelling, ≥12 months post-injury, traumatic (e.g., motor vehicle crash or fall) or non-traumatic/non-progressive SCI disease (e.g., spinal stenosis, infection, tumour) aetiology. Those with acute, sub-acute, congenital or neurodegenerative SCI, or severe cognitive impairment were excluded [32]. The present study used only data from participants ≥45 years of age.

### Data Collection

Data from the Aus-InSCI participants extracted comprised demographic and injury information (i.e., age, gender, residential location, household income, marital status, level and completeness of injury) and levels of participation in LTPA obtained with a modified version of the PASIPD [34]. The PASIPD provides information about people’s physical activities, including participation in LTPA and activities of daily living (ADLs), where individuals respond to 2 ordinally ranked responses. Frequency options range from 1 (never) to 4 (often) while duration options range from 1 (less than 1) hour to 4 (greater than 4 h), and a total score is usually reported in metabolic equivalent of task (MET) hours per day [34, 35]. The modified version of the PASIPD, used in the Aus-InSCI, asked participants how many days per week they participated in mild, moderate, and heavy LTPA, and how many minutes per day they performed those activities at each intensity, using a free text space, to provide a value of total LTPA (in minutes per week). These data were then categorised as never (0 days/week), seldom (1–2 days/week); sometimes (3–4 days/week); or often (5–7 days/week). For this study, we only used LTPA data, and excluded data on participation in ADLs.

### Data Analysis

Statistical analysis was completed in STATA-MP 17 software. Descriptive statistics were computed for sociodemographic and injury-related characteristics, as well as participation in total LTPA and moderate-to-heavy LTPA according to these characteristics.

#### LTPA

We used descriptive statistics to present the mean for total LTPA, moderate-to-heavy LTPA and strength-training LTPA, calculated in minutes per week. To deal with missing data on minutes per day of LTPA, we assumed that participants recording 0 days of participation in LTPA participated in 0 min of that type of LTPA. If the number of days of LTPA was missing, then minutes per day was also considered missing data. A total LTPA score combining light, moderate, heavy intensity, and strength LTPA days and minutes was calculated to determine each participant’s total LTPA volume in minutes per week (where no data was missing).

#### LTPA Versus Sociodemographic and Injury Characteristics

We used multivariable linear regression to estimate the association between the average minutes per week of participation in LTPA and the covariates age, gender, level and extent (completeness) of injury, time since injury, and cause of injury, based on inclusion in previous research [11, 36, 37] and the objectives of our study. Separate models were used for total LTPA, moderate-to-heavy LTPA and strength-training LTPA.

#### Dealing With Extreme Values and Missing Data

During initial data cleaning, 11 extreme observations (e.g., total LTPA >2,940 min per week, moderate-to-heavy LTPA >1,680 min per week) greater than the 99th percentile [38], were considered implausible and were excluded. The 11 extreme values were up to 4,620 min per week (equals 11 h per day) of total LTPA and up to 2,940 min per week (equals 7 h per day) of moderate-to-heavy LTPA.

In our statistical analysis, we evaluated patterns of missing data. We initially found a pattern of item non-response where participants reported “0” days of a type of LTPA without registering the number of minutes. We used a single-imputation strategy for these and imputed “0” into time where people had reported no days of that activity [39]. This improved the levels of missing data (Supplementary Figure S1) and we then used multiple imputation approach [deciding on 60 imputations after no change in estimated fraction of missing information (FMI)] with chained equations [40, 41] in a model with all LTPA day and time data, as well as auxiliary variables (Supplementary Table S1). Coefficients were across imputed datasets were combined with Rubin’s rules [42]. This analysis assumes data is “missing-at-random.”

#### LTPA Versus TSI

To investigate the association between LTPA and time since injury, a cubic spline model with four knots (at 5th, 35th, 65th, and 95th percentiles) was used as recommended [43], to allow the estimation between LTPA and the covariates without any assumption of the form of the relationship. Separate models were computed for total LTPA and moderate-to-heavy LTPA. To test the overall association of the spline form of TSI we used a likelihood ratio test.

## Results

Participants’ sociodemographic and injury characteristics are presented in Table 1. There were 1,281 participants included. The mean (SD) age was 62.7 (10.1) years, the mean (SD) time since injury was 18.7 (14.8) years, and 74% were male. Most participants sustained a traumatic injury (81.5%), had paraplegia (58.3%) and an incomplete injury (67%). The majority (54%) lived in metropolitan areas, which includes capital cities or other metropolitan centres (population > 100,000), with 25.1% living in rural centres (population 10,000–99,000), and 16.8% living in remote areas (population < 10,000). Most participants (63%) were married or in a partnership.

**TABLE 1:** Sociodemographic and injury characteristics in adults ≥45 years of age with spinal cord injury (n = 1,281) (Australia, 2024).

Characteristics	
Age (years), mean (±SD), median (IQR)	62.7 (±10.1) 62 (55–70)
Time since injury (years), mean (±SD), median (IQR)	18.7 (±14.8) 14 (6–30)
Sex, n (%) - Male - Female	944 (73.7) 337 (26.3)
Level of injury, n (%) - Paraplegia - Tetraplegia - missing	747 (58.3) 461 (36) 73 (5.7)
Severity of injury, n (%) - Complete - Incomplete - missing	391 (30.5) 858 (67) 32 (2.5)
Cause of Injury, n (%) - Traumatic - Non-traumatic - Unknown	1,044 (81.5) 226 (17.6) 11 (0.9)
Marital status, n (%) - Single - Married - Widowed - Separated/divorced - missing	227 (17.7) 808 (63.1) 62 (4.8) 182 (14.2) 2 (0.2)
Living place, n (%) - Capital city - Other metropolitan centres - Rural centres - Remote areas - missing	410 (32) 282 (22) 322 (25.1) 215 (16.8) 52 (4.1)
Household income, n (%) - Less than $455 per week - $455 - $686 - $687 - $909 per week - $910 - $1,203 per week - $1,204 - $2,374 per week - $2,375 or more per week - missing	303 (23.7) 165 (12.9) 138 (10.8) 109 (8.5) 209 (16.3) 180 (14.1) 1 (3.8)

SD, standard deviation; IQR, Interquartile range. Residential location: Capital city, Other metropolitan centres (population > 100,000), Rural centres (population 10,000–99,000), Remote areas (population < 10,000).

The amount and intensity of LTPA is presented in Table 2. Overall, 43.8% of participants reported no participation (zero minutes per week) in total LTPA, 74.9% reported no participation in moderate-to-heavy LTPA, and 52% reported no participation in strength-training LTPA. The average participation in total weekly LTPA was 197 (SD 352) minutes/week, and the median (IQR) was 50 (IQR 0–240) minutes per week. The participation in moderate-to-heavy LTPA average was 62 (SD 174) minutes/week, with a median of 0 (IQR 0–2) minutes/week. The average participation in strength-training was 63 (SD 123) minutes per week and the median (IQR) was 0 (IQR 0–75) minutes per week.

**TABLE 2:** Descriptive statistics (minutes per week) as a function of sociodemographic and injury characteristics of adults ≥ 45 years with spinal cord injury (Australia, 2024).

	LTPA (minutes per week)								
	Total			Moderate-to- heavy			Strength training		
	Mean ± SD	Median	IQR	Mean ± SD	Median	IQR	Mean ± SD	Median	IQR
Total sample (n = 1,281)	197 ± 352	50	0, 240	62 ± 174	0	0, 2	63 ± 123	0	0, 75
Sex									
- Male	214 ± 381	60	0, 270	67 ± 191	0	0, 12.5	69 ± 131	0	0, 90
- Female	148 ± 244	35	0, 210	42 ± 111	0	0, 0	47 ± 96	0	0, 60
Level of injury									
- Paraplegia	192 ± 343	50	0, 242	61 ± 167	0	0, 20	62 ± 117	0	0, 90
- Tetraplegia	212 ± 374	60	0, 270	67 ± 188	0	0, 0	66 ± 130	0	0, 70
Severity of injury									
- Complete	183 ± 340	30	0, 242	67 ± 182	0	0, 30	61 ± 127	0	0, 70
- Incomplete	205 ± 360	60	0, 240	60 ± 171	0	0, 0	64 ± 121	0	0, 80
Cause of injury									
- Traumatic	213 ± 367	60	0, 280	68 ± 182	0	0, 30	67 ± 129	0	0, 80
- Non-traumatic	122 ± 253	0	0, 140	35 ± 117	0	0, 0	45 ± 85	0	0, 60
Household income									
- Less than $455 per week	173 ± 375	0	0, 180	50 ± 155	0	0, 0	66 ± 146	0	0, 60
- $455 - $686	195 ± 373	60	0, 215	70 ± 199	0	0, 30	51 ± 102	0	0, 60
- $687 - $909 per week	185 ± 273	60	0, 315	55 ± 142	0	0, 0	59 ± 103	0	0, 100
- $910 - $1,203 per week	197 ± 321	70	0, 240	64 ± 204	0	0, 30	62 ± 92	30	0, 85
- $1,204 - $2,374 per week	278 ± 401	140	0, 400	95 ± 211	0	0, 120	77 ± 136	12	0, 120
- $2,375 or more per week	229 ± 376	150	0, 420	65 ± 169	0	0, 48	68 ± 124	0	0, 90
Residential location									
- Capital city	209 ± 335	60	0, 270	67 ± 181	0	0, 30	68 ± 119	0	0, 90
- Other metropolitan centres	194 ± 350	60	0, 220	67 ± 184	0	0, 40	62 ± 118	0	0, 80
- Rural centres	218 ± 389	60	0, 280	64 ± 168	0	0, 20	63 ± 132	0	0, 60
- Remote areas	191 ± 356	0	0, 210	57 ± 174	0	0, 0	57 ± 122	0	0, 70
Marital Status									
- Single	182 ± 323	35	0, 210	58 ± 173	0	0, 0	59 ± 109	0	0, 60
- Married/partnership	205 ± 352	60	0, 270	61 ± 170	0	0, 20	65 ± 123	0	0, 90
- Widowed	196 ± 401	30	0, 240	73 ± 225	0	0, 0	66 ± 141	0	0, 75
- Separated/divorced	185 ± 368	0	0, 240	70 ± 175	0	0, 30	59 ± 130	0	0, 60

LTPA, Leisure-time Physical Activity; SD, standard deviation; IQR, Interquartile range. The interquartile range column shows the 25th and 75th percentiles. All values in this table are untransformed but have excluded participants (n = 11) in the 99th percentile. Residential location: Capital city, Other metropolitan centres (population > 100,000), Rural centres (population 10,000–99,000), Remote areas (population < 10,000).

The multivariable regression analysis for total time spent in LTPA is presented in Table 3. Female gender (β = −62.3, 95% CI [−112.9, −11.7]), longer time since injury (β = −2.3, 95% CI [−3.9, −0.8]), and a non-traumatic cause of injury (β = −105.2, 95% CI [−165.9, −44.6]) were associated with less total time spent undertaking LTPA. Older age (β = −1.2, 95% CI [−2.4, −0.1]) and non-traumatic cause of injury (β = −32.5, 95% CI [−63.7, −1.4]) were associated with less moderate-to-heavy LTPA (Table 3). Female gender (β = −17.9, 95% CI [−34.2, −1.6]), longer time since injury (β = −1.0, 95% CI [−1.5,-0.5]) and non-traumatic cause of injury (β = −23.6, 95% CI [−43.4, −3.7]) were associated with less time performing strength-training (Table 3).

**TABLE 3:** Multivariable regression analysis for total time spent in leisure-time physical activity, with beta cofficient and 95% confidence interval for each type of leisure-time physical activity (Australia, 2024).

Covariate	Reference category	Total LTPA	Moderate-heavy LTPA	Strength training
Complete injury	Incomplete injury	−33.7 (−84.6; 17.1)	0.0 (−26.2; 26.3)	0.3 (−15.9; 16.6)
Tetraplegia	Paraplegia	−13.4 (−60.8; 34.1)	−7.8 (−32.5; 16.9)	−0.2 (−15.7; 15.3)
Non-traumatic injury	Traumatic injury	−105.2 (−165.9; −44.6)*	−32.5 (−63.7; −1.4)*	−23.6 (−43.4; −3.7)*
Age	/year	−1.3 (−3.6; 0.9)	−1.2 (−2.4; −0.1)*	0.6 (−0.1; 1.4)
Time Since Injury	/year	−2.3 (−3.9; −0.8)*	−0.3 (−1.1; 0.5)	−1.0 (−1.5; −0.5)*
Female Gender	Male	−62.3 (−112.9; −11.7)*	−25.3 (−51.1; 0.4)	−17.9 (−34.2; −1.6)*

*significant association where *p* < 0.05.

**FIGURE 1:**
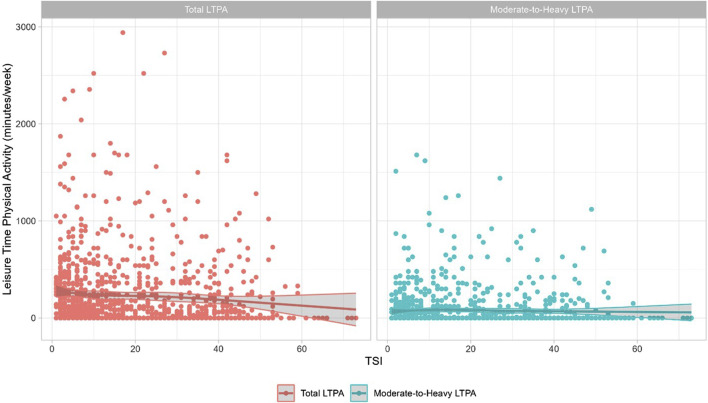
Leisure-time physical activity and time since injury with fitted cubic spline. Total Leisure-time physical activity: The relationship between total leisure-time physical activity (minutes/week) and time since injury (years) was significant for each participant (red-filled circles). Data were modelled using spine regression with four knots (five intervals) to investigate a non-linear relationship (red line). The spline prediction and likelihood ratio demonstrate there is limited evidence that time since injury (in spline form) was associated with total leisure-time physical activity (Probability > F = 0.0506). The shaded areas demonstrate 95% confidence intervals for the regression spline, which are more variable at higher time since injury. Moderate-to-Heavy leisure-time physical activity: The relationship between total moderate-to-heavy leisure-time physical activity (minutes/week) and time since injury (years) for each participant (blue-filled circles). Data were modelled using spine regression using four knots (five intervals) to investigate a non-linear relationship (blue line). The spline prediction and likelihood ratio demonstrate that time since injury (in spline form) is not associated with moderate-to-heavy leisure-time physical activity (Probability > F = 0.785). The shaded areas demonstrate 95% confidence intervals for the regression spline, which are more variable at higher time since injury (Australia, 2024).

We found a statistically significant linear and negative association between total LTPA time and time since injury (Figure 1). Further analysis of the spline prediction and the likelihood ratios did not find a clear association between variables (Probability > F = 0.0506) (Figure 1). We did not find an association between moderate-to-heavy LTPA and time since injury when analysing the spline prediction and likelihood ratio (Probability > F = 0.785) (Figure 1). Overall the maximum FMI in each regression model and overall relative increase in variance were moderate (Supplementary Table S2).

## Discussion

We undertook secondary analyses of an extensive cross-sectional survey that investigated levels of LTPA participation in Australians with SCI of all ages. The present study described the amount and intensity of LTPA participation in adults with SCI ≥45 years and assessed possible associations between LTPA participation and participants’ characteristics. While the averages for total LTPA participation and moderate-to-heavy LTPA appeared high, data were right-skewed, and the medians were very low. Three-quarters of the participants did not engage in any moderate-to-heavy LTPA and half did not engage in strength-training, meaning that a large proportion did not meet either the WHO or SCI-specific physical activity guideline recommendations [13, 14].

Our study highlights the importance of implementing early LTPA strategies for ageing women and those with non-traumatic injuries to mitigate the increased risk of reduced participation and health consequences. Despite statistically significant associations between total LTPA and time since injury, the B-Coefficients and 95% confidence intervals were small. We found limited evidence to suggest a clinically meaningful association between total LTPA and time since injury in the ≥45 years population, which differs from previous research in the general SCI population, which has suggested that those with longer time since injury (6–15 years) were more likely to be active than those injured for less than 5 years [3], and as injury duration progressed there was a increased chance that a person would be an exerciser [44]. Furthermore, this study found no evidence to suggest an association between participation in moderate-to-heavy LTPA and strength-training LTPA with time since injury, and that other personal and socioeconomic factors may be more important to identify to target those at risk of declining LTPA participation in people ≥45 years.

We found large variability in the reported time spent on total and moderate-to-heavy LTPA. This highlights the potential for some individuals with SCI ≥45 years of age to participate in long-term LTPA and maintain an active lifestyle, provided they can overcome the many psychosocial and environmental barriers to physical activity experienced in community [5, 45]. In people of all ages, the Aus-InSCI survey [44] suggested that so long as people begin LTPA post-injury, they were more likely to meet the physical activity volume recommendations. This potentially highlights that an important solution could be the development of strategies to embed LTPA into people’s lives early after injury [44].

There are differences when comparing LTPA participation in the SCI population to the non-disabled population. In the current investigation, 43.8% of participants reported no participation in LTPA, and 75% did not participate in the moderate-to-heavy intensity required to achieve health and fitness benefits. The Australian “45 and Up” study surveyed over 100,000 individuals from the general population and identified that three-quarters of respondents met the WHO physical activity recommendations [46]. Another large cross-sectional study conducted in the SCI population in Thailand found that only 14% of individuals met the SCI-specific physical activity guidelines and only 7% met the recommended WHO physical activity guidelines for adults with a disability [47], compared to 48% without a disability [14]. This confirms that a large proportion of individuals ≥45 years with SCI in Australia have a sedentary lifestyle compared to those who are non-disabled. As such, they are at increased risk of developing cardiometabolic diseases and raising the risk of poor health and wellbeing outcomes as they age [22, 48, 49]. Health promotion strategies must be explicitly tailored to adults ≥45 years with SCI, including programs and resources to promote accessible and enjoyable LTPA opportunities in the community.

The present Australian cohort showed some similarities with earlier work, which found that females with SCI have lower levels of LTPA participation [11, 50, 51]. In Sweden, it was suggested that traditional gender roles might be a barrier to access to LTPA for women [11]. Other sub-populations with low levels of LTPA (median of 0 min per week total and moderate-to-heavy LTPA) included low-income earners (<$455 per week), being separated or divorced, those living in remote areas (population < 10,000) and having non-traumatic injuries. There is the potential that adults ≥45 years with SCI living in regional and rural communities in Australia face unique geographic challenges due to the country’s size. Other key factors influencing LTPA participation in individuals with SCI include socioeconomic status. Our study revealed that those in the lower family income bracket engaged in the least LTPA. This underscores the significance of policymakers addressing barriers such as costs associated with gym attendance and sporting groups, purchasing specialised equipment and transportation expenses to enhance access and participation.

While cardiovascular fitness and health are necessary, mental health, wellbeing and quality of life outcomes are equally important for those ≥45 years with SCI. In general, people with SCI are 5% more likely to develop anxiety, 20% more likely to develop depression and 15% more likely to develop psychological issues than people without SCI [52]. However, older adults with long-term SCI report a low prevalence of depression, with one-third displaying clinically relevant depressive symptoms but only 5% displaying probable clinical depression [53]. Participation in LTPA has been shown to reduce anxiety and depression significantly and to improve the quality of life in those with long-term disabilities [21, 54]. Social exclusion can play an important factor in aging, especially for those with a disability [55], and LTPA participation could be a way for those aging with SCI to socialise, potentially improving mental health and quality of life. In summary, LTPA participation can significantly promote successful aging and overall health and quality of life for this population [21, 54].

### Strengths and Limitations

This study is the first to evaluate LTPA participation in adults ≥45 years of age with SCI in Australia. Our sample (1,281 people) provides an adequate representation of the Australian population living with SCI (approximately 20,800 people) [56] in the community as it included a large number of Australians from different states and territories.

A limitation of this study was the missing data on LTPA participation, which might have affected the generalisability of the findings. Further, some participants reported excessive levels of LTPA (up to 11 h per day). The extreme ranges of the LTPA data raise questions about the accuracy of the self-reported data. A drawback was that there was no way to “validate” the survey responses in a self-reported design. One potential factor contributing to these inconsistencies could be recall bias, which is associated with self-reported assessments of LTPA, leading to over-estimating activity levels [57]. The length of the Aus-InSCI survey was up to 1-hour and could have also contributed to survey fatigue, prompting some individuals to leave the LTPA questionnaire unanswered. Alternatively, participants could have deliberately misreported their responses for various reasons. Moreover, the confusion between ADL’s and LTPA may have led respondents to score general daily activities as LTPA if they perceived them as having sufficient physical stress due to their potentially limited mobility. This response bias could have resulted from a misunderstanding of what constitutes LTPA, impacting the accuracy of reported activity levels. Given that the Aus-InSCI was a survey, using objective measures such as accelerometers was not feasible. Another limitation is that the data collected was pre-COVID 19 pandemic, therefore a more current analysis on levels of physical activity in this population is warranted due to the potential impact of the restrictions related to the pandemic on physical activity behaviours. There has been minimal research conducted post the COVID-19 pandemic on levels of physical activity in people with SCI, however, one study determined that individuals with paraplegia who were full-time manual wheelchair users displayed lower levels of physical activity during the pandemic than in the pre-pandemic period [58]. We acknowledge that immediately post-pandemic LTPA participation may have changed due to decreased access to recreational and sports facilities or fear of contracting the virus in the community [59].

### Clinical Applications and Recommendations

Our findings demonstrated the importance of health promotion strategies to increase LTPA uptake among those ≥45 years with SCI, especially aging women, those with non-traumatic injuries, and those living in remote communities or with a lower socioeconomic status. To optimise LTPA engagement, we recommend the development of tailored LTPA community programs for older women and individuals with non-traumatic injuries, as they share increased susceptibility to inactive lifestyles. Health promotion strategies should include health professionals and clinicians providing education on the physical and mental health benefits of LTPA and offering information resources on local LTPA opportunities available upon discharge and within the community to maximize uptake [60]. Furthermore, extending physical activity opportunities to rural and remote regions via telehealth-exercise delivery in groups can enhance accessibility and community participation [61]. Moreover, the Australian National Disability Insurance Scheme (NDIS) and public and private sector insurers should support middle-aged and older people with SCI to access community based LTPA by providing, for example, gym memberships and funding for community sporting clubs or activities to ensure inclusivity and better health outcomes in this population.

We propose that future research should prioritise consumer-informed strategies to improve LTPA participation. By addressing inactivity in this population, we hope to minimise its health and financial burden. Further research could lead to developing new interventions and policy changes concerning LTPA, focusing on promoting healthy aging with SCI in our community from as early as 45 years. Clinicians and consumer agencies must educate these at-risk groups, which may reduce the strain on the healthcare system as the population of older people with SCI grows.

### Conclusion

We found considerable variation in the weekly time spent in LTPA in older individuals with SCI in Australia, with a large proportion (44%) not participating in any LTPA, and the majority (almost 75%) not participating in moderate-to-heavy LTPA. Ageing women and those with non-traumatic injuries tend to participate less in weekly LTPA. Moreover, time since injury was not associated with LTPA participation in our sample. While some participants with SCI can maintain an active lifestyle, many are not achieving the recommended dosage of physical activity that translates into health benefits and reduction of cardiometabolic disease risk.
