# Supplementary material for: Leisure-Time Physical Activity Participation in Middle-Aged and Older Adults With a Spinal Cord Injury in Australia
Source: Int J Public Health. 2024 Jul 3;69:1607276. doi: 10.3389/ijph.2024.1607276 (PMC11251882; doi:10.3389/ijph.2024.1607276)
Supplement: Supplementary file 1 [file DataSheet1.docx]

**Supplementary Material**

Supplementary Figure 1. Patterns of missing data for self-reported days and minutes of different categories of leisure time physical activity after initial imputation. Each row shows a unique pattern of missing variables with the number of missing cases with that pattern on the left axis.


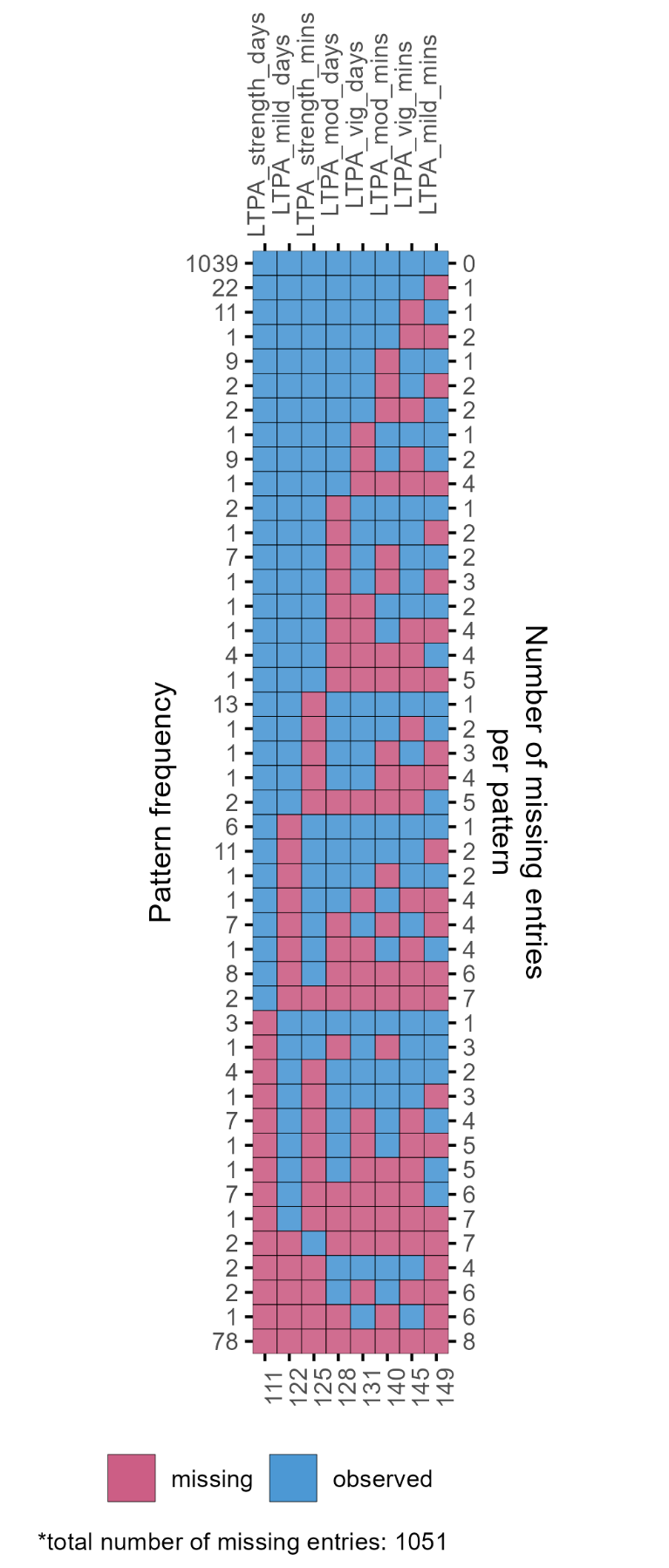


Supplementary Table 1. List of variables (auxiliary variables in **bold**) and imputation method in chained equations.

| Method |  |
| --- | --- |
| Predictive mean-matching (from 5 neighbours) | Age LTPA_mild_mins LTPA_mod_mins LTPA_vig_mins LTPA_strength_mins LTPA_mild_days LTPA_mod_days LTPA_vig_days LTPA_strength_days TSI |
| Binary logistic regression | Gender toi coi MOI |
| Multinomial logistic regression | **Living_place_rural_metro** |

Supplementary Table 2. The largest fraction of missing information (FMI) is from non-response and relative variance increases from non-response.

| Outcome | Largest FMI | RVI |
| --- | --- | --- |
| LTPA Total | 0.11 | 0.09 |
| LTPA moderate-to-heavy | 0.12 | 0.12 |
| LTPA strength | 0.12 | 0.17 |
